# Recent Advances in Zika Virus Vaccines

**DOI:** 10.3390/v10110631

**Published:** 2018-11-14

**Authors:** Himanshu Garg, Tugba Mehmetoglu-Gurbuz, Anjali Joshi

**Affiliations:** Center of Emphasis in Infectious Diseases, Department of Biomedical Science, Texas Tech University Health Sciences Center, El Paso, TX 79905, USA; tugba.gurbuz@ttuhsc.edu

**Keywords:** Zika virus, flaviviruses, vaccines, virus like particles, clinical trials, ZIKV

## Abstract

The recent outbreaks of Zika virus (ZIKV) infections and associated microcephaly in newborns has resulted in an unprecedented effort by researchers to target this virus. Significant advances have been made in developing vaccine candidates, treatment strategies and diagnostic assays in a relatively short period of time. Being a preventable disease, the first line of defense against ZIKV would be to vaccinate the highly susceptible target population, especially pregnant women. Along those lines, several vaccine candidates including purified inactivated virus (PIV), live attenuated virus (LAV), virus like particles (VLP), DNA, modified RNA, viral vectors and subunit vaccines have been in the pipeline with several advancing to clinical trials. As the primary objective of Zika vaccination is the prevention of vertical transmission of the virus to the unborn fetus, the safety and efficacy requirements for this vaccine remain unique when compared to other diseases. This review will discuss these recent advances in the field of Zika vaccine development.

## 1. Introduction

Zika virus (ZIKV) is a flavivirus that was first isolated in 1947 form a sentinel rhesus monkey in Uganda [1]. Since the identification of this new member of the *Flaviviridae* family, the virus has been implicated in several localized outbreaks. Currently, this mosquito borne flavivirus has been reported to be circulating in 26 countries and territories in Latin America and the Caribbean [2,3]. The natural transmission cycle of the virus involves vectors mainly from the *Aedes* genus, with monkeys as intermediate hosts and humans as occasional hosts [2]. Clinical symptoms of ZIKV infection include asymptomatic cases to an influenza like syndrome with fever, headache, malaise and cutaneous rash [4,5]. However recently, more cases of ZIKV in pregnant women with fetal microcephaly, a range of disorders referred to as congenital Zika syndrome (CZS), and other neurological disorders such as the Guillain–Barre syndrome have been described [6]. The sudden appearance of fetal defects associated with the current outbreaks has drawn international attention, with the primary objective of developing effective vaccines and therapeutics to combat the virus.

## 2. ZIKV Genomic Organization

**Figure 1:**
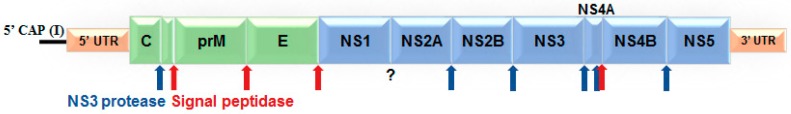
Zika virus genome. The viral genome comprises of a positive sense RNA that encodes a polyprotein that is processed by viral and host cell proteases. The amino terminus of the genome encodes the structural proteins (C-prM-E) essential for virion morphogenesis. The non-structural proteins NS1–NS5 are important for virus replication, polyprotein processing and invoking a cell mediated immune response, along with immune evasion.

The ZIKV genome consists of a single stranded positive sense RNA of 10.749 Kb. The genome consists of a single open reading frame encoding a polyprotein 5′-C-PrM-E-NS1-NS2A-NS2B-NS3-NS4A-NS4B-NS5-3′ [7,8] (Figure 1). This polyprotein is then cleaved into capsid (C), precursor of membrane (prM), envelope (E) and seven non-structural proteins. The E protein (~53 Kd) is the major surface protein involved in the virus binding to the cell surface and membrane fusion [8,9]. As the E protein is the primary target for neutralizing antibodies, it is the preferred antigen in most vaccine platforms. Expression of the structural proteins (prME) of most flaviviruses is sufficient for the formation of sub-viral particles (SVPs) which makes these proteins prime candidates for vaccine development [10,11,12,13]. SVPs share several properties with wild-type viruses, such as fusogenic activity [14] and the induction of a neutralizing antibody response [12,13]. The flaviviral capsid protein is important for encapsidation of the viral genome along with interactions with lipids for particle formation [15]. While presence of the capsid protein is not required for the generation of sub-viral particles, inclusion of the protein has proven to be beneficial for vaccine purposes, for a better cell mediated immune response [16]. The flaviviral nonstructural proteins are important for several key aspects of virus replication. With regards to virus assembly, NS3 is the key protein that cleaves the terminal capsid and prME via its N terminal region in conjunction with NS2B, known as NS2B3 [10]. The non-structural proteins also help in evasion of the host’s innate immune response by inhibiting production of anti-viral molecules, like the interferons [17].

## 3. Zika Disease Outbreak and Pathogenesis

The recent outbreaks of ZIKV were highly concerning due to the devastating outcome of microcephaly and other birth defects in the unborn fetus. Although identified in 1947, ZIKV remained obscure, circulating in certain African and Asian countries with limited number of reported cases. The 2007 outbreak in the Yap island, Micronesia, was the first outbreak beyond Africa and Asia followed by the 2013 outbreak in the French Polynesia with 11% of the population seeking medical attention. Soon thereafter, Brazil saw one of the worst outbreaks of ZIKV in 2015 with some areas reporting sero-positivity as high as 60% [18]. While the recent virus outbreaks have clearly been associated with marked fetal defects and congenital Zika syndrome, the reason for the sudden appearance of these defects, particularly with the current outbreak, remains unknown. Several theories have been proposed for the phenomenon including infection of neuronal progenitor stem cells during pregnancy [19], enhancement of infection due to pre-existing antibodies to Dengue virus (DENV) and/or West Nile virus (WNV) [20], presence of mutations in the envelope glycoprotein of the Asian lineage [21], environmental factors, etc. The incidence of microcephaly during the 2015 ZIKV outbreak was ~100 fold higher than the baseline microcephaly cases in the US, providing the first evidence of a link between Zika infection and birth defects. [22]. Furthermore, the isolation of ZIKV from placental tissue and infected fetuses directly linked the virus to the birth defects, although it remains uncertain if the virus alone is sufficient to induce congenital brain defects [23]. A recent study by Reynolds et al., found that in the United States, 15% of pregnant women with confirmed ZIKV infection in the first trimester had babies with Zika-associated birth defects [22].

## 4. Need for a Vaccine

Currently there are no ZIKV specific antivirals approved for clinical use, making prophylactic vaccination the best approach to combat the disease. As of now, control of mosquito vectors, prophylaxis against mosquito bites and prevention of pregnancy in endemic areas are the only feasible prophylaxis measures available to reduce the risk of fetal transmission. Although the mortality rate for ZIKV infections is low, the incidence of Guillain Barré syndrome following ZIKV infection warrants vaccination in endemic areas. More importantly, the high incidence of ZIKV induced fetal development disorders like microcephaly in pregnant women suggests an urgent need for an effective vaccine. Although the WHO has declared that the ZIKV outbreak is no longer a Public Health Emergency of International Concern (PHEIC), due to a decline in the number of cases seen in 2018, the risk of a new outbreak in the near or distant future cannot be ignored. As per the WHO Target Product Profile (TPP) for ZIKV vaccines, the immunization of women of reproductive age, including pregnant women, is the highest priority. Various models have suggested that prioritizing ZIKV vaccination in women of child bearing age is likely to control ZIKV mediated prenatal infections and Congenital Zika Syndrome (CZS) [24]. A ZIKV vaccine that is safe for use in pregnant women is needed not only to combat an outbreak like the recent one in Brazil, but also for routine vaccination in endemic areas [25] that propagate/sustain the mosquito vectors imperative for virus transmission.

## 5. Characteristics of a Zika Vaccine

Experience from vaccines against other flaviviruses like Japanese Encephalitis Virus (JEV) and Yellow Fever Virus (YFV), suggest that a vaccine against ZIKV will need to fulfill unique criteria. As per Centers for Disease Control (CDC) guidelines, the Purified Inactivated Virus (PIV) vaccine against JEV and the live attenuated vaccine against YFV are largely contraindicated, in pregnant women unless the benefits outweigh the risk. As the target population of the ZIKV vaccine is largely women who are or may become pregnant, the vaccine will need to meet high safety standards. While safety remains a top priority, the efficacy of the ZIKV vaccine also needs to be high as vertical transmission of virus during pregnancy is associated with CZS. Although sterilizing immunity is unlikely to be achieved by a flaviviral vaccine, candidates that elicit a strong enough immune response to prevent trans-placental transfer of the virus to the fetus would be desirable. However, it is unclear what titer of neutralizing antibodies will achieve this, although results from vaccine studies in mouse model show promising results [26]. As ZIKV infections are spreading largely in developing and under developed countries, the cost of vaccination will also be a key consideration point for mass vaccination campaigns. Thus, it would be safe to say that any platform for a ZIKV vaccine must overcome the safety, efficacy and economical barriers to be successful.

## 6. Zika Vaccine Development

The response by the research community to the recent Zika outbreak has been unprecedented. In less than three years, multiple vaccine candidates have gone through preclinical testing and several have made it to clinical trials (Table 1). The vaccine candidates currently being pursued are discussed below.

### 6.1. DNA Vaccines

DNA vaccines against ZIKV were amongst the first platform to be developed concurrently by several groups [26]. All of these approaches utilize the expression of prME proteins from ZIKV, as expression of these proteins in mammalian cells leads to assembly of sub-viral particles that are non-infectious, but retain structure and antigenicity similar to native virions. Larocca et al. were the first to demonstrate that a DNA vaccine expressing codon optimized prME from Brazil (BeH815744 strain, Accession # KU365780) was immunogenic and protected mice against a virus challenge [27]. Similarly, Dowd et al. developed a DNA vaccine incorporating the prME proteins (French Polynesian strain H/PF/2013, Accession # KJ776791) which was immunogenic and provided protection against viremia in mice, as well as non-human primates [28]. Similar studies were conducted by Muthumani et al. who also showed that a DNA vaccine generated via a synthetic prME construct was protective in mice and non-human primates [29]. Additionally, in this study IFNAR−/− mice that were highly susceptible to Zika infection, were protected after DNA vaccination. These vaccine platforms have now progressed to clinical trials (Table 1) and preliminary safety data shows that the DNA vaccines are safe and effective. Gaudinski et al. [30] tested the safety and efficacy of two DNA vaccines; VRC 5288 that expresses a chimeric E protein containing Zika and Japanese encephalitis sequences, and VRC 5283 that expresses the wild type Zika E protein. In both vaccines, the prM signal sequence comprised of the JEV signal to improve particle secretion. In phase 1 clinical trials, the neutralizing response to VRC 5283 was superior to VRC 5288 with 100% of the participants showing neutralizing antibodies in the split needle-free dose group, making it a better candidate to progress to phase 2 clinical trials. Similarly, Tebas et al. [31] tested the efficacy of a Zika DNA vaccine (GLS5700) expressing prME regions of ZIKV from a synthetic construct derived from a consensus sequence. The vaccine showed development of neutralizing antibodies in 63% of the participants. Other DNA platforms have also progressed to clinical trials and are listed in Table 1. Although DNA vaccines provide many advantages like ease of production and rapid adaptation to new and emerging infectious agents, a head to head comparison of DNA vaccine with Purified Inactivated Virus (PIV) vaccine showed that PIV vaccines fare better in terms of immunogenicity, at least in the rhesus monkeys [32].

### 6.2. Purified Inactivated Virus (PIV) Vaccines

Larocca et al. described the use of a PIV ZIKV vaccine in June 2016, immediately after the 2015 outbreaks [27]. They used the Puerto Rican strain (PRVABC59) for the vaccine candidate that was passaged in Vero cells followed by inactivation with formalin. The alum adjuvanated vaccine provided complete protection in mice after a single immunization followed by challenge with ZIKV-BR (Brazil ZKV2015). Interestingly, higher antibody titers were achieved in the intramuscular versus subcutaneous immunized mice that also correlated with better protection after challenge. In the same study, the authors also compared a DNA based prME vaccine which protected mice completely against ZIKV challenge, after a single immunization. Simultaneously, in September 2016, the same group [32] evaluated the efficacy of the PIV vaccine platform against ZIKV in rhesus monkeys which provided complete protection against virus challenge. The PRVABC59 isolate was used in two doses (weeks 0 and 4) in conjunction with alum and protected monkeys against challenge with the Brazilian and Puerto Rico ZIKV isolate. Moreover, passive transfer of antibodies from immunized monkeys protected both mice and monkeys from ZIKV challenge, in a dose dependent manner. Two doses of the formalin inactivated PRVABC59 vaccine provided protection in monkeys even when challenged a year after immunization [33]. This was superior to two immunizations with a DNA counterpart that conferred protection early at the peak of antibody titers, but efficacy against challenge declined after a year.

The Walter Reed Army Institute conducted three phase 1 trials [34] of the above PIV vaccine using alum adjuvant (*N* = 55) and placebo controls (*N* = 12). The objective was to test the safety and efficacy of the PIV candidate in humans. Healthy participants were administered two doses of the PIV vaccine 29 days apart which was well tolerated with minimal side effects like pain, tenderness at the injection site or generalized fatigue. The neutralizing antibody titers obtained were ≥1:10 at day 43 and adoptive transfer of purified IgG from immunized recipients into Balb/c mice offered protection after challenge evident by reduced viral loads. Sumathy et al. developed a PIV vaccine using the African ZIKV isolate (MR766) and formalin inactivation [35]. Two doses of the vaccine provided 100% protection against the homotypic and heterotypic (FSS 13025, Accession #KU955593) ZIKV strains in AG129 mice. Moreover, passive transfer of immune sera generated in rabbits protected Balb/c mice against live virus challenge. On a related note, Yang et al. [36] described a cDNA clone of PRVABC59 with mutations in the NS1 (K265E) prME (H83R) and NS3 (S356F) proteins that increased virus yield by more than 25 fold when compared to the WT clone. This would not only facilitate high titer virus yield for PIV production in less time, but also minimize the introduction of unwanted mutations upon continuous virus passage.

### 6.3. Live Attenuated Virus (LAV) Vaccines

Experience from vaccination studies with other flaviviruses like YFV and JEV suggest that live attenuated vaccines (LAV) for ZIKV are likely to be effective. The most widely used LAV for YFV is derived from the 17D strain that has been in use since 1937, and shows significant protection [37]. Similarly, the JEV vaccine based on the attenuated SA14-14-2 strain has been extensively used in endemic areas [38]. With regards to ZIKV, Shan et al. [39] developed a LAV vaccine by deleting 10 nucleotides in the 3′ untranslated region (UTR) of a Cambodian strain of ZIKV (FSS 13025, Accession #KU955593). This attenuated virus provided sterilizing immunity with saturating neutralizing antibody titers in WT adult mice, and did not induce pathology after intracranial injection in 1 day old immunocompetent mice. A single dose of the vaccine also protected rhesus macaques against viremia upon challenge. Further analysis of this LAV vaccine showed that a single dose significantly reduced vertical transmission of Zika in pregnant mice, and prevented testicular damage in male mice [40]. Zou et al. [41] developed a DNA launched version of the LAV vaccine with a 20 bp deletion in the 3′-UTR, and demonstrated the efficacy of the vaccine at extremely low doses (0.5 μg), compared to other DNA vaccines. Besides the direct attenuation of ZIKV, other approaches like chimeric viruses have been used for making attenuated vaccines. Xie et al. [42] showed that a chimeric virus containing Zika prME in a DENV-2 backbone was highly attenuated in A129 mice and provided protective immunity against Zika challenge. Similarly Li et al. [43] used the attenuated JEV strain SA14-14-2 as the backbone to introduce the Zika prME region of an Asian ZIKV strain FSS 13025. When administered as a single dose, this vaccine provided protection against placental and fetal damage in pregnant mice and protected the offspring against intracranial virus challenge. A single vaccine dose was also protective in rhesus monkeys that were challenged with the virus.

### 6.4. Virus like Particles (VLP) Vaccines

In case of flaviviruses, expression of the structural proteins (prME or CprME) gives rise to non-infectious virus like particles that resemble infectious virions. This provides a method for presentation of viral antigens in a highly native conformation. Thus, VLP vaccines often surpass the elicited immune response when compared to subunit vaccines that are comprised of single proteins [44]. Moreover, VLPs present antigens in their native conformation in a repetitive manner that is recognized effectively by B cells leading to their activation and production of high titers of specific antibodies. Another advantage of VLP vaccines is their ease of production and scalability providing an economic platform, especially by generation of stable cell lines. Due to their non-infectious nature, VLPs are safe for administration in immunocompromised adults, children and pregnant women.

Surprisingly, with regards to ZIKV, the description of VLP platforms came much later than the PIV, DNA and mRNA counterparts. Garg et al. [45] reported the development of prME and CprME VLPs (Suriname 2015, Accession # KU312312.1) and compared them to their DNA counterparts in efficacy studies in mice. Immunization with CprME VLPs generated higher neutralizing antibody titers than prME VLPs. DNA vaccination with prME was less effective than VLPs with CprME DNA, failing to generate neutralizing antibodies in mice. An advantage of the approach by Garg et al. was the generation of stable cell line secreting the prME VLPs, thereby eliminating the need for routine transfections and DNA amplification for large scale VLP production. This study also brought forth the advantage of incorporating the capsid protein in the VLPs for an enhanced immune response. In a similar study, Boigard et al. [46] compared CprME VLPs (Strain H/PF/2013, Accession # KJ776791.1) with formalin inactivated ZIKV (PIV) and found superior antibody generation with CprME VLPs, versus inactivated virus. While using a transient transfection protocol for VLP generation, their study emphasized the deleterious effects of chemical inactivation on neutralizing epitopes for vaccine production.

Thereafter, a number of studies described the use of different VLP platforms for ZIKV. Yang et al [47] developed a VLP displaying the EIII domain of ZIKV (PRVABC59 (amino acids 1–403, Accession # AMC13911)) on the Hepatitis B core antigen. The advantage of this system was the easy production and purification from Nicotiana plants. The vaccine elicited a strong neutralizing and cellular immune response against different ZIKV strains, and the elicited antibodies did not cause enhancement of Dengue virus infection in cells expressing the Fcγ receptor. Espinoza et al. [48] also developed a prME VLP vaccine ((prM sequence from the African MR766 strain (Accession # KU955594); the E ectodomain from the Brazilian SPH2015 strain (Accession # KU321639) and the stem-anchor from the African MR766 strain (Accession # KU955594)) containing a heterologous IL-2 signal sequence and found that the immune sera generated in WT CB6F1 mice was protective of ZIKV infection in AG129 mice. Basu et al. [49] displayed highly immunogenic potential B cell epitopes from the E protein of ZIKV (strain MR-766) on bacteriophage particles, and saw moderate protection in challenge studies in Balb/c mice. Baculovirus expressed Zika VLPs [50] displaying the prME proteins (strain SZ-WIV01) elicited a potent neutralizing and T cell response in immunized mice. Salvo et al. [51] used the Zika CPrME (strain H/PF/2013, Accession # KJ776791) VLPs to immunize Balb/c and AG129 mice followed by challenge studies and observed decreased viremia or increased survival in respective models.

### 6.5. Modified mRNA Vaccines

mRNA technology has recently gained popularity in the field of vaccination as mRNA’s pose minimal risk of integration into the host genome making them safer than DNA vaccines. mRNA vaccines make use of the host cell processes to translate viral proteins and generally encode the protein of interest, untranslated regions at the 5′ and 3′ end give stability in cells and replacement of uridine residues in the sequence with other natural modifications, prevent indiscriminate innate immune activation [52,53]. Moreover, encapsulation of naked mRNA into lipids not only increases stability, but also helps in high protein expression by aiding in intramuscular delivery. After the 2015 ZIKV outbreaks, one of the first vaccine strategies to be described was the use of modified mRNA to protect against ZIKV infection in two simultaneous reports published in March 2017 [54,55]. Richner et al. developed a modified RNA with a type-1 cap, a signal sequence from human IgE or JEV, and the prME genes of ZIKV Asian strain (Micronesia 2007, Accession # EU545988). The modified RNA was encapsulated in lipid nanoparticles to aid in intramuscular delivery and showed excellent protection in WT C57BL/6 and AG129 mice upon virus challenge leading to sterilizing immunity. The authors also tested the role of Antibody dependent enhancement (ADE) inducing epitopes in the DII region of the flavivirus E protein by generating modified mRNA vaccines containing mutations in the E-DII-FL region. Interestingly, they showed that mutations of the DII-Fusion loop epitope diminished generation of antibodies that would enhance Dengue virus infection. The same group [56] also showed protection against “ZIKV induced congenital disease”, after vaccination of pregnant dams with the lipid nanoparticle vaccine, containing the modified mRNA followed by virus challenge. Similarly, Pardi et al. [54] simultaneously demonstrated that immunization with a modified mRNA encoding the prME of ZIKV (H/PF/2013, Accession # KJ776791) encapsulated into lipid nanoparticles was able to confer potent B and T cell responses in mice and strong ZIKV specific neutralizing antibody titers in non-human primates, after a single immunization. Moreover, low doses of the vaccine (30 µg in mice and 50 µg in non-human primates) was sufficient to protect the animals against live virus challenge. Thereafter Chahal et al. [57] developed a modified dendrimer nanoparticle (MDNP)-RNA vaccine that expressed the prME proteins of ZIKV (Accession # KU312312, Asian lineage virus isolated from a patient in Suriname in 2015) cloned into an RNA replicon vector. Their approach identified a 9 amino acid long highly conserved MHC-1 restricted epitope in the E protein capable of inducing a CD8 specific T cell response in mice.

Soon after the reports of ZIKV mRNA vaccines, Moderna therapeutics in collaboration with Biomedical Advanced Research and Development Authority (BARDA) initiated clinical trials of the vaccine in 2016. The objective was to “evaluate the safety and immunogenicity of mRNA 1325 Zika vaccine in healthy adults in a non-endemic Zika region”, via a phase 1, randomized, and placebo controlled trial. The study is set to complete in February 2019, enrolling 90 participants to assess any adverse events and seroconversion when compared to placebo controls. The Phase 1 study was funded by BARDA with $8 million, which will pave way for Phase 2 and 3 clinical studies with $117 million, along with supporting large scale manufacturing of the vaccine for worldwide dissemination.

### 6.6. Subunit Vaccines

As the E protein of flaviviruses is the major antigen for generation of neutralizing response, this protein has been used in various forms as a subunit vaccine candidate for DENV [58] and WNV [59]. Building on the same premise, To et al. [60] expressed and purified soluble ZIKV E protein produced in Drosophila melanogaster cells. The expression vector contained an insect cell optimized synthetic gene, expressing the entire prM and amino acids 1–408 of the E protein from the French Polynesian strain (Accession # KJ776791). Immunization of mice with purified recombinant E protein in combination with adjuvant resulted in neutralizing antibody titers that were protective against viremia in immunocompetent mice. Tai et al. [61] fused the ZIKV E domain III region to the C terminal Fc region of human IgG, to generate a recombinant immunogen. This recombinant E protein contained amino acids 298–409 of ZIKV E (ZIKV SPH2015 Accession # KU321639.1) and was found to be highly immunogenic, resulting in neutralizing antibody generation and protection of mice in various models. Yang et al. [62] developed a subunit vaccine derived from the EIII domain of strain PRVABC59 produced as *E. coli* inclusion bodies. The vaccine evoked a high titer neutralizing antibody response that did not enhance DENV infection in vitro, along with production of Th1 and Th2 cytokines by splenocytes. The same group [63] also described a plant produced subunit E vaccine that was found to be highly immunogenic, as seen via neutralizing antibody and T cell responses. Qu et al [64] described ZIKV subunit vaccines produced in insect cells and comprised of the domain III or the 80% N terminal region of the E protein (Accession # KU312312). Both vaccines generated antibody and T cell specific responses with the domain III vaccine being superior. Recently, Zhu et al [65] described a subunit vaccine comprising of the N terminus (450 amino acids) of the E glycoprotein that was capable of inducing long term protection in mice. Immunization of pregnant dams with the subunit vaccine also protected the fetal brains in utero and neonates against microcephaly.

### 6.7. Viral Vectors

Using viral vectors to deliver antigens for the purpose of developing immunity is a new and emerging field. Adenoviruses and vaccinia virus are perhaps the most well studied and widely used platforms for vaccine development. Replication deficient versions of these viruses are safe and potent inducers of immunity against antigens of choice. Abbink et al. [32] used rhesus Adenovirus 52 (RhAd52) as vector expressing ZIKV prME in rhesus monkeys. In the same study they compared multiple vaccine platforms and found that a RhAd52-prME vaccine generated neutralizing antibodies, and complete protection from challenge in rhesus monkeys after a single immunization. Xu et al. [66] developed a recombinant chimpanzee adenovirus type 7 (AdC7) expressing ZIKV M and E proteins from the Asian linage of ZIKV FSS13025 (Accession # MH158236), isolated in Cambodia. In mouse models of ZIKV, a single immunization protected the mice against ZIKV viremia and testicular damage after challenge. Similarly, Cox et al [67] developed an Adenovirus serotype 26 (Ad26) based vaccine containing the ME region of ZIKV strain BeH815744 (Accession # KU365780.1). Single immunization with this adenoviral vaccine provided protection against challenge in both mice and Non-human primates (NHP) models. To improve upon the antigenicity of ZIKV adenoviral vaccines, Lopez-Camacho et al. [68] modified the prME region (consensus sequence) by deleting the transmembrane (TM) region of Envelope (Env). This prMEΔTM region in a chimpanzee adenoviral vector (ChAdOx1) provided better neutralizing response than the full length prME, suggesting that modification of antigen membrane anchor can enhance immunogenicity. Liu et al. [69] recently included the NS1 gene of ZIKV along with prME (isolate 1_0080_PF, Accession # KX447521) in an Adenovirus serotype 2 vector system to generate Ad2-prME-NS1 vector. The Ad2-prME-NS1 vector resulted in both Env as well as NS1 specific antibodies, and protected pups born to immunized dams against Zika induced pathology.

Recently, Prow et al. [70] incorporated ZIKV prME (Brazilian isolate SPH2015, Accession # KU321639) as well as a Chikungunya virus (CHIKV) structural protein cassette into a vaccinia derived Sementis Copenhagen Vector (SCV) system. This multi pathogen vaccine provided protection against both ZIKV and CHIKV in wild type and IFNAR−/− mice. Development of a similar multivalent vaccine against arboviruses that circulate in the same geographical region may have added advantages. Betancourt et al [71] were the first group to use the recombinant Vesicular stomatitis virus (VSV) platform to express either Zika Env or Zika prME proteins from a SPH2015 isolate (Accession # KU321639). Two immunizations with these vectors resulted in an anti Zika antibody response that protected newborn mice born to vaccinated mothers against lethal Zika challenge. Emanuel et al. [72] recently used a recombinant VSV that expressed the codon optimized prME (Accession # KU681081.3) region of Zika along with an Ebola GP protein. This vector was built upon the previous Ebola vaccine vector, VSV-EBOV vaccine [73,74]. The vector was tested in IFNAR−/− mice and provided protection from lethal challenge after a single immunization as late as 3 days prior to ZIKV challenge. However, the replicating nature of the recombinant VSV vectors make them less likely to be used in vulnerable populations like pregnant mothers. The rapid nature of protection makes them ideal for controlling outbreaks in certain geographical regions.

## 7. Conclusions

There is no doubt that the response from the research community with respect to a Zika vaccine has been unprecedented. With the tremendous progress made in this field, a viable vaccine for ZIKV is both possible and closer than expected. However, the lack of new Zika cases in 2018 has dampened the enthusiasm as well as urgency for the vaccine. Moreover, lack of uniformity in the assays being used to determine vaccine efficacies makes comparison of different candidates difficult. The reduction in Zika cases also makes it difficult to study the efficacy of the vaccine in clinical trials. Moreover, conducting placebo control trials in pregnant women in Zika endemic areas has serious ethical issues. Nevertheless, a safe effective and economical vaccine for Zika should be developed in order to prepare for a subsequent outbreak which remains a real possibility. This lesson has already been learned from the overwhelming 2015 outbreaks, more than 50 years after the identification of the virus.

## Figures and Tables

**Table 1:** Zika vaccine platforms in clinical trials.

Clinical Trial No	Platform	Phase	Sponsor	Vaccine Name	Antigen	Dosage	Application	Intervals	N	Planned End Date
**NCT02840487**	DNA Vaccine	I	NIAID	VRC 319	prME	4 mg	Needle and syringe (Deltoid, IM)	0–8; 0–12; 0–4–8; 0–4–20 week	80–120	Dec 2018
**NCT02996461**	DNA Vaccine	I	NIAID	VRC 320	prME	4 mg	Single or Split-dose needle or PharmaJet (Deltoid, IM)	0–4–8 week	45	Dec 2018
**NCT03110770**	DNA Vaccine	II	NIAID	VRC 705	prME	4 or 8 mg	PharmaJet (Deltoid, IM)	0–4–8 week	2400	Jan 2020
**NCT02887482**	DNA Vaccine	I	Inovio Pharmaceuticals and GeneOne Life Sciences	GLS 5700	prME	2mg	CELLECTRA-3P electroporation (ID)	0–4–12 weeks	160	Jun 2018
**NCT02809443**	DNA Vaccine	I	Inovio Pharmaceuticals and GeneOne Life Sciences	GLS 5700	prME	1 or 2 mg	CELLECTRA-3P electroporation (ID)	0–4–12 weeks	40	Nov 2017
**NCT02963909**	Purified Inactivated Virus	I	Walter Reed Army Institute of Research and NIAID	ZPIV	Whole Virus	5 mcg	IXIARO(JEV) prime ZPIV+Alum or YF-VAX (YFV) prime ZPIV+Alum (IM)	0–4-ZPIV; 0–4 JEV-16–20 ZPIV; 0-YFV-12–16–ZPIV week	75	Feb 2019
**NCT02952833**	Purified Inactivated Virus	I	Walter Reed Army Institute of Research and NIAID	ZPIV	Whole Virus	2.5, 5.0 and 10 mcg	Injection of vaccine with Alum (IM)	0–4–12 weeks	91	Jun 2019
**NCT02937233**	Purified Inactivated Virus	I	Walter Reed Army Institute of Research and NIAID	ZPIV	Whole Virus	5 mcg	Injection of vaccine with Alum (IM)	0; 0–2; 0–4 week	36	Jun 2018
**NCT03008122**	Purified Inactivated Virus	I	Walter Reed Army Institute of Research and NIAID	ZPIV	Whole Virus	2.5 or 5 mcg	Injection of vaccine with Alum (IM)	0–4 week	90	Jan 2020
**NCT03425149**	Purified Inactivated Virus	I	Valneva	VLA1601	Whole Virus	3 or 6 AU (Antigen Units) of ZIKV	Injection of vaccine with Alum (IM)	0–1; 0–4 week	67	Nov 2018
**NCT02996890**	Live attenuated recombinant vaccine	I	Themis Bioscience	MV-ZIKA	prME in measles vector	High and Low dose	IM Injection	0; 0–4 week	48	Apr 2018
**NCT03014089**	mRNA vaccine	I/II	Moderna Therapeutics	mRNA-1325	prME	NA	NA	NA	90	Sep 2018
**NCT03343626**	Purified Inactivated Virus	I	Takeda	TAK-426	Whole Virus	2, 5, 10 mcg	Injection of vaccine with Alum (IM)	0, 4 week	240	Sep 2020
**NCT03611946**	Live Attenuated Virus	I	NIAID	rZIKV/D4Δ30-713	Whole genome	10^3^ plaque-forming units (PFU)	SC	0 week only	28	Sep 2019
**NA**	Purified Inactivated Virus	I	Bharat BioTech	MR 766	Whole Virus	5, 10 mcg	IM	0–4 week	48	Not Known

NIAID = National Institute of Allergy and Infectious Diseases; mcg = micrograms; NA = Not available; IM = Intramuscular; ID = Intradermal; SC = Subcutaneous.
